# Working Memory Load Attenuates Emotional Enhancement in Recognition Memory

**DOI:** 10.3389/fpsyg.2013.00112

**Published:** 2013-03-18

**Authors:** Ewa A. Miendlarzewska, Gijs van Elswijk, Carlo V. Cannistraci, Raymond van Ee

**Affiliations:** ^1^Department of Fundamental Neurosciences, Centre Médical Universitaire, University of GenevaGeneva, Switzerland; ^2^Sapiens Steering Brain Stimulation B.V.Eindhoven, Netherlands; ^3^Biological and Environmental Sciences and Engineering Division, Computer Electrical and Mathematical Sciences and Engineering Division, Computational Bioscience Research Center, Integrative Systems Biology Laboratory, King Abdullah University of Science and TechnologyThuwal, Kingdom of Saudi Arabia; ^4^Division of Medical Genetics, Department of Medicine, University of CaliforniaSan Diego, CA, USA; ^5^Laboratory of Experimental Psychology, Katholieke Universiteit LeuvenLeuven, Belgium

**Keywords:** emotional enhancement of memory, distraction, working memory, cognitive load, negative emotions

## Abstract

Emotionally arousing stimuli are perceived and remembered better than neutral stimuli. Under threat, this negativity bias is further increased. We investigated whether working memory (WM) load can attenuate incidental memory for emotional images. Two groups of participants performed the *N*-back task with two WM load levels. In one group, we induced anxiety using a threat of shock paradigm to increase attentional processing of negative information. During task performance we incidentally and briefly flashed emotional distracter images which prolonged response times in both load conditions. A subsequent unannounced immediate recognition memory test revealed that when load at exposure had been low, recognition was better for negative items in both participant groups. This enhancement, however, was attenuated under high load, leaving performance on neutral items unchanged regardless of the threat of shock manipulation. We conclude that both in threat and in normal states WM load at exposure can attenuate immediate emotional memory enhancement.

## Introduction

Frequently, emotional information enters our memory not when we are paying particular attention to it but due to incidental perception while busy with some primary task. Emotional signals are remembered better than neutral (LaBar and Cabeza, 2006) and it has been reported that threat-related stimuli can be processed in an automatic manner with minimal attention (Vuilleumier et al., 2001, 2002). Distracting visual arousing stimuli can be remembered involuntarily and bias our memory of an event that happened to contain an emotional distracter leading to, for instance, negative associations, and aversive behaviors (Baeyens et al., 2005), as well as false memories (Smeets et al., 2008). Anxiety can significantly modulate the degree of attention required for the processing of threat-related stimuli, which makes anxious individuals more prone to detect, and remember negative information (Fox, 2002). Thus, understanding how bias in incidental memory can be influenced is of interest for prevention of traumatic memories and anxiety disorders.

Various studies have explored the use of cognitive load to regulate negative mood, since it is known that restricting attentional resources can attenuate the emotional impact of negative stimuli (Erthal et al., 2005). Most of the data supports the theoretical proposal that negative emotional expression competes with high load task performance in the cognitive/attentional resource space (Pessoa, 2009; Kron et al., 2010; Shafer et al., 2012), such that anxiety and working memory (WM) performance are negatively correlated (Vytal et al., 2012). Many studies have pointed out this attentional capacity dependence: reported feelings are less intense when emotion induction takes place during concurrent task performance (Kron et al., 2010) or when a difficult task is administered right after negative picture viewing (Van Dillen and Koole, 2007). Distraction can also be used actively as an emotion regulation strategy (Mcrae et al., 2009), and negative mood can be replaced by cognitive task-related thoughts (Erk et al., 2007). Importantly, cognitive load can reduce subsequent negative memories and flashbacks when applied up to 4 h after a traumatic experience, but only certain tasks are effective in this respect (Holmes et al., 2010). Therefore, the investigation of the effects of cognitive load on emotional memory formation is very important not only as a means to regulate emotions but also in the context of affective disorders.

Amongst the unresolved issues of emotion-cognition interactions is the effect of emotional enhancement when memory is probed immediately after encoding. Specifically, it is unknown to what extent the memory-prioritization is due to increased attention and to what extent other processes, such as facilitated retrieval, memory consolidation or even increased false memory (Gallo et al., 2009), or judgment bias (Sharot et al., 2004; Phelps and Sharot, 2008) may play a role in this phenomenon.

Emotional enhancement of memory (EEM) observed in many paradigms appears to be driven at least partially by enhanced attentional processing (MacKay et al., 2004; Talmi et al., 2007), but at the same time EEM benefits from the reduction in task-irrelevant processing because it is being preserved even under divided-attention conditions (Kensinger and Corkin, 2004). In fact, negative emotional stimuli induce dual effects on cognitive control processes: on the one hand, they interfere with ongoing processing slowing down response times in tasks involving perceptual judgment or detection (Vuilleumier et al., 2001; Shafer et al., 2012; Talmi and McGarry, 2012), and on the other hand, when used as targets, they improve performance and conflict resolution due to reduction of task-irrelevant processing (e.g., Pessoa, 2009; Hu et al., 2012).

Previous studies have often neglected to control for spatial deployment of attention or used potentially ineffective distracters. For instance, one study has shown that perceptual load at exposure brings the recognition of fearful faces down to chance level (Jenkins et al., 2005). In this experiment, faces were presented as background to the letter detection task such that each trial consisted of a face and a superimposed letter string. Subjects were instructed to ignore the face and focus on the strings. One may argue that in this design, the face was part of the target display and not a distracter as its onset and presence were fully predictable. In another study testing short-term memory, when neutral and emotional words were presented very briefly so as to increase task difficulty, EEM was abolished (Sharot et al., 2004). Yet, attention was not controlled in that experiment leaving the question of the extent of attentional and WM resource involvement in EEM unanswered. A third problem is that previous studies used paradigms where participants are explicitly instructed to encode information (e.g., Talmi et al., 2007), which does not always correspond to real life situations where memories are often formed incidentally.

### This study

We tested two hypotheses based on the arousal-biased competition model of emotional memory (“ABC,” Mather and Sutherland, 2011) while addressing the shortcomings of the previous studies. First, we tested the effect of cognitive load on short-term emotional memory using distracters in a way that endows them with all attributes of bottom-up prioritization. Second, by inducing threat of electric shock in one of the experimental groups, we tested whether enhancing top-down prioritization related to the participants’ emotional state can change the competition and resulting memory.

In two groups of participants, we tested whether WM load is able to win the competition from bottom-up factors, namely sudden, unexpected, perceptually distinct, and emotionally arousing images presented centrally in the focus of attention. Our participants performed an *N*-back WM task with two load levels. Using an unannounced recognition memory test we assessed whether the level of WM load during initial exposure differentially influenced emotional memory enhancement for the distracters. In the second group of participants, we manipulated emotional relevance of negative images by introducing threat of electric shock, which should lead to anxiety and arousal (Robinson et al., 2011) and as a consequence, to prioritized processing of emotional state congruent stimuli (i.e., negative pictures). The shock-threat paradigm has been shown to reliably induce anxiety, especially if the shock is unpredictable (Grillon et al., 2004) and for that reason is frequently used to model behavior in anxiety disorders. Our experiment tests whether anxiety leads to a negativity bias in immediate recognition memory when encoding is unintentional and whether it persists when WM load is increased.

Emotional distracters’ efficacy was controlled by ensuring their sudden, unpredictable onset, and brief duration; spatial attention was maintained centrally fixed as distracters appeared on top of the task-relevant targets; and participants were instructed to concentrate on the primary task and ignore the distracters to minimize the variability of cognitive set (which is a top-down factor in the ABC model).

According to the arousal-biased competition model (Mather and Sutherland, 2011), increased arousal in the shock-anticipation condition could further increase the detection of the negative affective distracters. At the same time, this condition is of particular interest because arousal should enhance goal-directed processing thus inhibiting the effects of distraction on task performance (Schupp et al., 2003). Theory also predicts that if the arousing stimulus is not in direct competition with the task-relevant stimuli, then the processing of neutral goal-relevant items should be enhanced, and the processing of less relevant stimuli should be reduced. Based on this, two competing hypotheses can be drawn: either the participants under threat will show a relatively larger negativity bias in immediate recognition memory (as compared to the neutral experimental group), or they will show overall lower memory for all distracters due to the distracter-inhibition effect of fearful emotional state. In addition, while we expect to observe an EEM in the neutral group in the low load condition, the difference in recognition accuracy between negative and the remaining images may be larger in the group of participants tested under threat of shock. Arousal imposes a certain level of WM load which should lead to a general decrement in performance in the WM task, that would now compete for scarcer cognitive resources (Vytal et al., 2012). Based on the latter fact, as well as on the above-mentioned evidence of load-induced reduction of emotion processing, we expect that under high WM load, overall memory for distracters will be poorer in both groups, with a possibly larger effect in the threat group, and abolition of the EEM.

## Materials and Methods

### Participants

In both experimental groups, 21 naïve volunteers with normal or corrected-to-normal vision participated (group 1: mean age 23 years, SD = 2, 10 females; group 2: mean age 33 years, SD = 10, 4 females). The experiment was approved by the local ethics committee and written informed consent was obtained from every participant. To rule out the effects of prior memory, participants were screened for naivety: knowledge of experimental methods of cognitive psychology and previous participation in affective picture rating experiments were criteria for exclusion.

### Materials

In total, 144 affective color pictures were used in the experiment. Seventy-two were presented as distracters during the *N*-back task (24 per emotional category; 12 in each load condition) and as targets in the recognition memory test. The other 72 pictures were only used as decoys in the recognition memory test (24 of each emotional category). The majority of the images (48 negative, 48 positive, and 20 neutral) was taken from a validated existing set of affective pictures, that were tested by 86 independent raters on emotion and arousal scales, and were controlled for luminance differences (Overbeek et al., 2007). The negative pictures were taken from subsets “fear” and “anger” of the database in which the average ratings on the dimension arousal were, respectively *M* = 1.8 (*M* = 2.9 on unpleasantness) and *M* = 1.65 (*M* = 3.45 on unpleasantness) on a five-point scale. The positive pictures were drawn from subsets “positive surprise” and “enjoyment” in which average arousal was respectively *M* = 1.8 and *M* = 1.95 (and respectively, *M* = 2.45 and *M* = 2.95 on pleasantness). Please note that although on the face value these arousal ratings appear low, the images we selected were amongst the highest rated in the database. Our picture database was modeled after the IAPS (Lang et al., 2008), and picture ratings within the different emotional categories did not fundamentally differ from the IAPS ratings^1^. Negative images contained mutilated bodies, war images, sharks, snakes, and guns, etc. Positive images contained puppies, children, social celebrations, and landscapes. Neutral pictures depicted people in conversation, nature, and household objects. Twenty-eight new unrated pictures of objects and scenes were added to the neutral pictures (mainly images of household objects) to reach a total of 48 images per emotional category.

We performed a Shapiro–Wilk test on the arousal ratings in the three sets of images for which the ratings were available (negative/positive/neutral), according to which all of them are non-Gaussian distributed. Thus, we cross-compared the median of the three sets using a permutation test (which is an exact test), and adjusted the *p*-values for multiple testing with Bonferroni correction. None of the cross-comparisons (negative-positive-neutral) resulted in a statistically significant difference using the conventional *p*-value threshold of 0.05. Table 1 summarizes the arousal-pleasantness evaluation of the distracter images.

**Table 1:** **Mean arousal ratings of emotional pictures based on *N* = 86 independent responders on a five-point scale**.

	Arousal ratings		
	Negative (*n* = 48)	Positive (*n* = 48)	Neutral (*n* = 20)^a^
High Load	1.83 *(*SD ± 0.26)	1.99 (SD ± 0.53)	1.60 (SD ± 0.39)
Low Load	1.61 (SD ± 0.16)	1.93 (SD ± 0.58)	1.56 (SD ± 0.32)
Foils	1.58 (SD ± 0.21)	1.85 (SD ± 0.47)	

*^a^Ratings are available for *n* = 20 neutral images. In addition, 28 neutral images were used in the experiments for which independent ratings were not available*.

At 60 cm viewing distance the images measured between 3.7° × 4° and 3.7° × 6.3° in visual angle. Distracter pictures contained scenes with people, animals, everyday use objects, and sceneries but no faces in close-up.

### Procedure and task

The participant was informed that the purpose of the experiment was to examine cognitive performance in a WM task. The participant sat behind a table with a laptop computer (14 inch display; viewing distance ∼60 cm). The main tasks were WM tasks with two load levels. The low load (LL) condition was a 1-back task; the high load (HL) condition was a 2-back task. The *N*-back task is a commonly used task to engage the WM, in which subjects are presented with a continuous stream of items and instructed to press a key when a repetition at a specified delay of *N* occurs.

**Figure 1:**
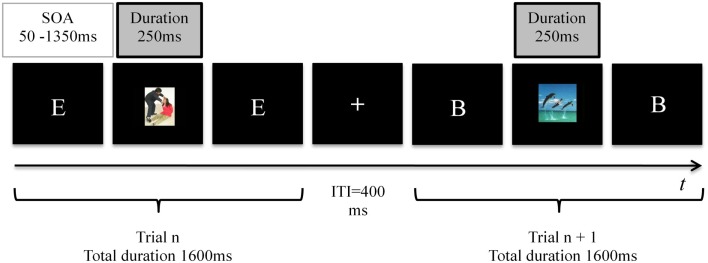
**Trial structure of the *N*-back tasks**. In the 1-back (low load) task a match was defined as the occurrence of two consecutive identical letters, whereas in 2-back (high load) task a match occurred when the presented letter was identical to the letter that had been presented one before the last. Subjects indicated matches and non-matches with button presses. The duration of each task was 144 trials (4 min 48 s). In 36 random trials an emotional distracter was presented for 250 ms, with a random SOA varying between 50 and 1350 ms.

A trial consisted of a white letter (A, B, C, D, or E; each letter measured ∼1.76° × 1.5° in visual angle) appearing for 1600 ms centrally on the computer screen on a black background, followed by a 400 ms blank black screen (Figure 1). Subjects were required to respond with a key press at every trial: Left-Alt for a match; right-Ctrl for a non-match. Letters were presented in random order and the proportion of match and non-match trials in each load condition was equal. In a random quarter (36) of the trials a distracter picture was briefly flashed centrally for 250 ms, covering the letter. SOA of the distracter was randomly varied between 50 and 1350 ms, to prevent anticipation. In both load conditions, 12 negative, 12 positive, and 12 neutral distracters were flashed. The distracter order was randomized between participants. The participant was requested to focus on accuracy in performance of the *N*-back task and to ignore the irrelevant distracters throughout. No feedback about correct or incorrect responses was provided before the end of the task. Each participant performed one test run per load level, and each run contained 144 trials (4 min 48 s). The order of load levels was balanced across subjects. In the middle of each run, a 10 s break was offered.

The *N*-back tasks were followed by an unannounced recognition memory test. Seventy-two new pictures (24 of each negative, positive, and neutral) were added to the set of previously presented distracters, totaling 144 images. The 144 images were displayed in sequence, in the center of the display on a black background. Subjects proceeded to the next picture by giving either of the two response options: when they recognized having seen the picture during the WM task (“seen”) or otherwise (“not seen”). Participants were informed beforehand that half of the images had been presented during the *N*-back tasks and that half was novel. The test was self-paced, and took ∼5–10 min. The images were presented in random order.

All tests were presented using Presentation software (Version 14.5, 2010 Neurobehavioral Systems, Albany, CA, USA).

### Induction of threat

Experimental group 2 performed the same task as group 1 while under continuous threat of a (sham) electric shock (Chua et al., 1999). The anxiety induction procedure was specifically implemented to improve attentional processing of the negative images (Fox, 2002) and thus memory thereof.

At the beginning of the session the participant was informed that the purpose of the experiment was to examine cognitive performance under threat. After the participant was seated behind the monitor, two ECG gel electrodes were placed on the right wrist. These electrodes were connected to a sham shock-delivery apparatus. The sham device was lying on the table in sight of the participant and remained switched on, indicated by a visible green led. The participant was told that a single electric shock would be delivered via the wrist electrodes. This would occur once at a random moment within the entire duration of the WM tasks. The participant was informed that the shock could be perceived as slightly painful, but was nonetheless entirely harmless. Because electric shocks can be a powerful confounding variable, our manipulation was limited to a threat (Laretzaki et al., 2011). No actual electric stimuli were delivered at any time. The electrode was removed right after the *N*-back task has been completed so that the participants knew that no shocks could be delivered during the subsequent memory test. The participants were thoroughly debriefed at the end of experiment, with particular consideration on the sham electrical stimulation.

### Validation of anxiety induction

In the group that underwent the threat manipulation, at the end of the experimental session, each participant indicated their level of anxiety during the experiment on a Likert scale from 1 (not at all anxious) to 5 (very anxious). After this check, the participants were thoroughly debriefed.

### Data analysis

Of the data from the *N*-back tasks, the first three trials of each participant in each block were regarded as warm-up trials and were excluded from the analyses. Responses with latencies of 150 ms or less were considered to be impulsive and were excluded from the analysis. For analysis of response times on distracter trials, only correct responses on those trials with distracters appearing >100 ms after letter onset were selected. This criterion was applied to avoid the possibility that a participant having missed the first, very quick appearance of the letter had to wait until the distracter disappeared in order to recognize the letter, which would artificially prolong response times on distracter trials. In addition, to eliminate the trials on which it was suspected that the distracter had no effect on the response, trials with reaction times (RT) occurring earlier than 100 ms after distracter onset were excluded from analysis. This selection retained ∼95% of all original trials. Using the remaining dataset the error rate and average RT were computed for each participant and condition.

From the data of the recognition memory test, we determined the hit rate (proportion of correctly identified distracters that had been shown during the *N*-back task) for each of the six conditions (2 load levels × 3 emotional categories). In order to compute the enhancement in memory due to emotion, average hit rates for each participant in neutral images were subtracted from negative and positive images, respectively. Thus obtained scores (referred to as EEM) were entered into a mixed-model 3-way repeated measures ANOVA with two independent repeated factors emotion (negative, positive) and load (low, high); and one between-subjects factor group (threat, no-threat). Also, for each of the three emotional image categories, we determined the false alarm rate (decoys falsely identified as seen during the *N*-back task). Subsequently we computed the *corrected recognition* scores (hit rate – false alarm rate) for each of the six conditions per participant.

Hit rate was computed as $Hit\;rate=\frac{\#hits}{\#hits+\#misses},$ and false alarm rate as $FA\;rate\;=\;\frac{\#false\;alarms}{\#false\;alarms\;+\;\#correct\;rejections}$.

Since the foils have never been presented before, the same false alarms rate (different for negative, positive, and neutral images) was used to compute the recognition rate in both load conditions. Although a corrected recognition score of −1 is possible theoretically (classifying all distracters as “unseen” while false alarming on all decoys), in practice the corrected scores should range between 0 (pure guessing) and 1 (correctly identifying all without any false alarms).

When considering the EEM, one must note the controversy surrounding the origin of this phenomenon. Several influential studies have pointed that emotion enhances the “feeling of remembering” (e.g., Sharot et al., 2004; Rimmele et al., 2011) rather than memory accuracy *per se*. In addition, methodological considerations suggest that the modeling of the underlying processes of recollection and familiarity is not yet clear (e.g., the single- vs. dual-process hypothesis, Yonelinas et al., 2010). Some studies reporting “EEM” claim that such enhancement is not due to increased recollection but rather enhanced feeling of remembering accompanying emotional, and especially negative items (Sharot et al., 2004), or that it originates from higher semantic relatedness amongst emotional items (Talmi et al., 2007). An alternative account focuses on the model of the memory recollection process as expressed by the signal detection theory, diffusely used in psychology. This account suggests that stronger subjective feeling of remembering leads to a more liberal bias to judge emotional items as remembered without an increase in sensitivity (Dougal and Rotello, 2007). To assess whether this is the case in our experiment, we computed a measure of response bias – the criterion location c.

The measure of response bias is derived from signal detection theory and is calculated as follows:

$c=-\frac{1}{2}\left[z\left(H\right)+z\left(F\right)\right],$ where *H* and *F* are hit- and false alarm rates, respectively (MacMillan and Creelman, 2005). This measure was calculated for all subjects for each of the 6 conditions.

Reaction times and error rates were analyzed each using a mixed-model 3-way repeated measures ANOVA (rANOVA) with independent repeated factors load (low, high) and trial type (regular, distracter), and a between-subjects factor Group (threat, no-threat). Corrected recognition scores, hit rates, as well as criterion location measure *c*, were the dependent variables analyzed each using the same mixed-model rANOVA, in which the two independent repeated factors were Emotion (negative, positive, neutral) and Load (low, high); and the between-subjects factor was Group (threat, no-threat). False alarm rates were analyzed with a mixed-model 2-way rANOVA with a repeated factor emotion (negative, positive, neutral) and a between-subjects factor group.

The analyses were followed by pair-wise comparisons if appropriate, and applying Bonferroni correction for multiple-comparisons where necessary. Levene’s test of equality of variances and Box’s *M* test of homogeneity of covariances were controlled for within-subjects and between-subjects effects, respectively, in all statistical analyses. A threshold of *p* < 0.05 for Levene’s and *p* < 0.001 for Box’s *M*, as recommended in Meyers et al. (2005), were used to determine whether the assumptions of the mixed-model were violated. All statistical analyses were performed using SPSS Statistics Release 18 (IBM, Somers, NY, USA). In all reported rANOVAs Greenhouse-Geisser corrections for violation of the sphericity assumption were applied before determining the significance levels. To aid readability the uncorrected degrees of freedom are reported.

## Results

### Emotional manipulation verification

The mean measure of anxiety during the *N*-back tasks obtained in *N* = 21 subjects in the shock-threat manipulation group (group 2), was *M* = 2.76 (SD ± 1.18) on a five-point Likert scale where 1 indicated “not anxious” and 5 indicated “very anxious.” Based on these reports we may conclude that the participants were on average moderately affected by the fear induction.

### *N*-back task performance

**Figure 2:**
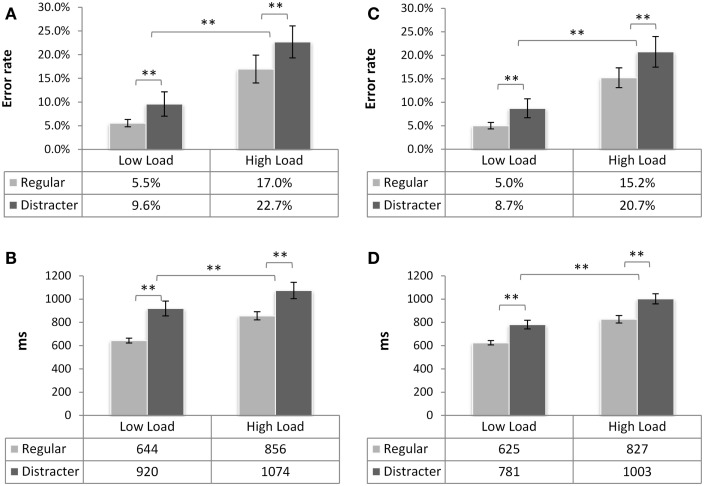
***N*-back task performance of in experimental group 1 (neutral, left) and group 2 (under threat, right)**. Mean error rate and mean reaction time (both ± SE of mean) as a function of working memory load and of trial type presented for group in neutral emotional state **(A,B)**, and under threat **(C,D)**. Statistical differences are denoted by **(*p* < 0.01). No significant difference between the groups was found (*p* > 0.1 for RTs, *p* > 0.6 for errors). For all graphs, the table underneath specifies the mean scores per condition, across participants.

Average error rates and RTs for each condition can be found in Figure 2,^2^. The statistical analysis revealed the main effect of load [*F*(1,37) = 69.9, *p* < 0.001, $\eta_{p}^{\text{2}}=0\text{.65}$] with longer RTs on HL trials, and a main effect of trial type [*F*(1,37) = 43.7, *p* < 0.001, $\eta_{p}^{\text{2}}\text{ = 0}\text{.54}$] such that responses were slower on distracter trials. No main effect of group was discovered [*F*(1,37) = 2.45, *p* = 0.126, ns.] nor any interactions between the factors [load × trial type *F*(1,37) = 0.001, *p* = 0.97, ns.; load × group *F*(1,37) = 1.15, *p* = 0.29, ns.; trial type × group *F*(1,37) = 2.7, *p* = 0.108, ns.].

Error rates in both groups were higher in the HL condition [*F*(1,37) = 43.08, *p* < 0.001, $\eta_{p}^{2}=0.54$] and significantly higher on distracter trials than on regular ones [*F*(1,37) = 17, *p* < 0.001,$\eta_{p}^{\text{2}}\text{ = 0}\text{.315}$]. The groups did not differ in the amount of errors [*F*(1,37) = 0.274, *p* > 0.6, ns.] and there were no interactions involving factors load or trial type (all *p*s > 0.4). Thus, load was successfully manipulated and distracters significantly interfered with performance in the *N*-back task.

As this analysis showed that distracters significantly slowed RTs, we investigated whether this interference depended on the valence of the distracter. Response times on these trials were analyzed with the mixed-model, including repeated factors load and emotion, and a between-subjects factor group. As the main RT analysis, this analysis showed that responses were faster in the LL condition (*M* = 851 ms, SE = 37 ms) than in HL [*M* = 1026 ms, SE = 48 ms; *F*(1,37) = 14.11, *p* < 0.001, $\eta_{p}^{2}=0.276$]. Importantly, neither the main effect of emotional category [*F*(2,74) = 0.204, *p* = 0.736] nor any interactions involving factors Group and Emotion were significant [all *p*s > 0.36, ns.]. Although we noted that the experimental group performing the *N*-back task under threat was somewhat faster to respond on the distracter trials (*M* = 882.7 ms, SE = 52 ms, neutral group *M* = 994.6 ms, SE = 54 ms), the main effect of group did not reach the significance threshold [*F*(1,37) = 2.23, *p* = 0.143, ns.]. In sum, presentation of a distracter resulted in longer response latencies at both load levels but no differences due to emotional valence were detected. *N*-back task performance in the group under threat was comparable to that of the group without emotion induction.

### Recognition memory accuracy

Hit rates for each condition are displayed in Table 2,^3^. The data revealed a strong main effect of Emotion with highest hit rates for negative images [*F*(2,76) = 10.87, *p* < 0.001, $\eta_{p}^{\text{2}}\text{ = 0}\text{.22}$], a main effect of Load with higher scores in the LL condition [*F*(1,38) = 20.95, *p* < 0.001, $\eta_{p}^{\text{2}}\text{ = 0}\text{.355}$] a main effect of Group with overall lower scores in the threat group [*F*(1,38) = 4.6, *p* = 0.038,], and an interaction Load × Emotion [*F*(2,76) = 15, *p* < 0.001, $\eta_{p}^{\text{2}}\text{ = 0}\text{.28}$]. There were no interactions involving the factor Group (all *p*s > 0.28).

**Table 2:** **Hit rates in experimental groups 1 (neutral) and 2 (under threat of electric shock)**.

		Hit rates		
		Negative	Positive	Neutral
Group 1 (neutral)	Low load	0.51 (±0.05)	0.42 (±0.05)	0.31 (±0.05)
	High load	0.29 (±0.04)	0.30 (±0.04)	0.35 (±0.05)
Group 2 (threat)	Low load	0.44 (±0.05)	0.27 (±0.05)	0.21 (±0.04)
	High load	0.23 (±0.05)	0.20 (±0.04)	0.20 (±0.04)

*Values in brackets indicate SE of the Mean*.

Next, the analysis was split along the factor Emotion in order to analyze the interaction effect. This analysis resulted in a main effect of Load in negative images [*F*(1,38) = 32.26, *p* < 0.001,$\eta_{p}^{2}=0.46$], and positive images [*F*(1,38) = 10.39, *p* = 0.003, $\eta_{p}^{2}=0.215$] but no such effect in case of neutral images [*F*(1,38) = 0.003, *p* = 0.66, ns.]. The main effect of Group was significant in all but in case of negative images [positive *F*(1,38) = 4.88, *p* = 0.033, $\eta_{p}^{2}=0.11;$ neutral *F*(1,38) = 5.7, *p* = 0.022, $\eta_{p}^{2}=0.13$; negative *F*(1,38) = 1.39, *p* = 0.25], suggesting that the reduction in hit rate due to WM load was similar for both experimental groups for negative images, while the between-group difference persisted under HL for positive and neutral images (with better scores in the neutral group).

The analysis of the EEM scores revealed a highly significant effect of load [*F*(1,38) = 24.6, *p* < 0.001, $\eta_{p}^{2}=0.39$] with a higher EEM in LL [*M*(LL) = 0.149, SE ± 0.027; *M*(HL) = −0.017, SE ± 0.022], as well as emotion [*F*(1,38) = 8.56, *p* = 0.006, $\eta_{p}^{2}=0.18$], and an interaction load × emotion [*F*(1,38) = 7.39, *p* = 0.01, $\eta_{p}^{\text{2}}\text{ = 0}\text{.16}$]. There was no significant effect of group [*F*(1,38) = 0.74, *p* = 0.39, ns.] and no significant interactions involving the factor group (all *p*s > 0.19). Although not statistically significant, it may be of interest that the planned comparisons indicated a stronger negative EEM in the threat group [*M* = 0.13, SE ± 0.03] than in the group with no emotion induction [*M* = 0.069, SE ± 0.031], but the two groups did not differ in the positive EEM (*M* = 0.032). The interaction was followed-up by paired *t*-tests comparing the negative and positive EEM in each load condition. This showed that the negative EEM was significantly larger than the positive EEM in LL [*t*(39) = 4.16, *p* < 0.001] but not in HL [*t*(39) = 0.29, *p* > 0.7, ns.]. One-sample *t*-tests also confirmed that the EEMs in low load were different from 0 [negative *t*(39) = 6.7, *p* < 0.001; positive *t*(39) = 2.83, *p* < 0.01], in contrast to the HL condition, where the EEM was abolished [negative *t*(39) = −0.36, *p* > 0.7; positive *t(*39) = −0.81, *p* > 0.4].

The results of the analysis of false alarm rates (Table 3) revealed a main effect of emotional category with higher FA rates for emotional items [*F*(2,76) = 7.475, *p* = 0.003, $\eta_{p}^{\text{2}}\text{ = 0}\text{.164}$] and no main effect of group [*F*(1,38) = 2.04, *p* = 0.16, ns.] and no interaction group × emotion (*p* > 0.95), suggesting that the nominal ratio of negative false memories was no higher in the participants under threat than in those in a neutral state.

**Table 3:** **False alarm rates in Experimental Groups 1 (neutral) and 2 (under threat of electric shock)**.

	False alarm rates		
	Negative	Positive	Neutral
Group 1 (neutral)	0.21 (±0.05)	0.19 (±0.04)	0.13 (±0.04)
Group 2 (threat)	0.15 (±0.04)	0.13 (±0.03)	0.07 (±0.02)

*Values in brackets indicate SE of the Mean*.

**Figure 3:**
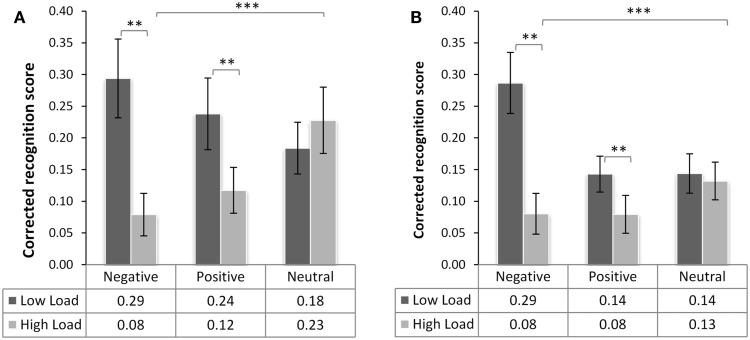
**Recognition memory performance of the neutral (A) and threat group (B)**. Mean *corrected recognition* score (±SE of mean) as a function of emotional category and the level of working memory (WM) load during initial presentation of the distracter. The table underneath the graph specifies the mean scores across participants per condition. There were significant main effects of WM load and interaction of load and emotional category (***p < 0.001). There was no significant difference between the groups (*p* > 0.2, ns.). WM load significantly reduced the performance for negative and positive but not neutral distracters (**p < 0.01).

In the analysis of the corrected recognition scores from the memory test (Figure 3), we found a main effect of load [*F*(1,38) = 20.81, *p* < 0.001, $\eta_{p}^{2}=0.35$], confirming that recognition was lower for images displayed in the HL condition, but no main effect of emotional category [*F*(2,76) = 1.375, *p* = 0.26, $\eta_{p}^{2}=0.35$]. There was, however, a significant interaction of load × emotional category [*F*(2,76) = 15.04, *p* < 0.001, $\eta_{p}^{2}=0.28$]. We did not find any interactions involving the factor Group [group × load *F*(1,38) < 0.08, *p* = 0.95, $\eta_{p}^{2}<0.0001;$ group × valence *F*(2,76) = 1.16, *p*.318, $\eta_{p}^{2}=0.03$], nor a triple interaction between group type, valence, and load. Even though the recognition accuracy in the non-threat group was slightly higher than in the threat group [in LL *M* = 0.24 (SD = 0.24) vs. *M* = 0.19 (SD = 0.18) in threat group; and in HL *M* = *0.14* (SD = *0.19*) vs. *M* = 0.10 (SD = 0.14), respectively], this effect was statistically not significant [main effect of group *F*(1,38) = 1.38, *p* = 0.246, ns.].

Splitting this overall ANOVA across the factor “emotional category,” revealed that the load × emotional category interaction was driven by the difference in recognition of emotional images between the two load levels [negative HL-LL *t*(39) = 5.73, *p* < 0.001 and positive HL-LL *t*(39) = 3.18, *p* = 0.009], while load had no effect on recognition accuracy of neutral pictures [*t*(39) = −0.526, *p* = 0.602]. From this result we may conclude that the two groups do not differ in recognition memory accuracy for emotional distracters presented during high and low WM load and that load leads to elimination of the memory advantage for emotional but not neutral images.

Calculation of *c* for all subjects in this experiment showed that they were most liberal, i.e., most likely to respond “seen” to negative images followed by positive, and that they were most conservative in recognizing the neutral images (results reported in Table 4). In the neutral group, the subjects were generally conservative in their answers showing a bias to respond “no” most of the time (57% in LL and 67.5% in HL) even though they had been instructed that they had seen 50% of the images during the task. In the threat group, the participants were very conservative in their responses responding “unseen” 70% of the time upon viewing pictures presented in low- and 80% in HL condition (detailed results in Table 4 below).

**Table 4:** **Measure of response bias derived from signal detection theory: mean criterion location measure *c* in recognition memory responses for experimental groups 1 and 2**.

		Criterion location *c*		
		Negative	Positive	Neutral
Group 1 (neutral)	Low load	0.48 (±0.12)	0.65 (±0.11)	0.99 (±0.13)
	High load	0.79 (±0.13)	0.82 (±0.12)	0.93 (±0.13)
Group 2 (threat)	Low load	0.75 (±0.13)	1.08 (±0.15)	1.33 (±0.11)
	High load	1.12 (±0.16)	1.21 (±0.14)	1.37 (±0.11)

*Higher values indicate more conservative responding. Group 1 (no emotion induction) *N* = 19 participants; Group 2 (threat of electric shock) *N* = 21. Values in brackets indicate SE of the Mean*.

Previous research on emotional words suggests that response bias shifts toward most liberal judgment of items as “seen” when the words were negative, less so for positive, and most conservative for neutral (Dougal and Rotello, 2007, Exp. 1A). This led us to suspect that we might find a similar bias in our experimental groups. A recent study using the same emotion induction paradigm as used here, reported in addition an annihilation of positivity bias (Robinson et al., 2011), which is another expected observation that the measure *c* will provide in characterizing the between-group differences in this study. Specifically, we expected to observe this effect in a group × emotion interaction in response bias.

Analyzing the response bias measure *c* (reported in Table 4), we discovered a main effect of group [*F*(1,38) = 5.25, *p* = 0.028, $\eta_{p}^{2}=0.12$] with the experimental group under threat of shock having a stronger bias to respond “no” (more conservative). This tendency was confirmed by the higher hit rates in the neutral group. We further found highly significant main effects of load [*F*(1,38) = 18,71, *p* < 0.001, $\eta_{p}^{2}=0.33$] with more conservative responses in HL, and a main effect of emotion [*F*(2,76) = 15.97, *p* < 0.001,$\eta_{p}^{2}=0.29$] with highest (most liberal) *c* values for negative, followed by positive and then neutral images, and an interaction between factors load and emotion [*F*(2,76) = 14.9, *p* < 0.001, $\eta_{p}^{2}=0.28$]. No interactions involving factor group were observed [all *p*s > 0.58]. Splitting the analysis along the factor emotion revealed that increased load did not decrease the response bias criterion only in the case of neutral images [no main effect of load *F*(1,38) = 0.062, *p* = 0.8, *ns*], while it did have an effect on negative [*F*(1,38) = 31.86, *p* < 0.001, $\eta_{p}^{2}=0.456$] and positive images [*F*(1,38) = 9.79, *p* = 0.003, $\eta_{p}^{2}=0.20$]. This result echoes the findings in corrected recognition rates. Conversely, similarly to the hit rates analysis, the two groups of participants were significantly different in terms of response bias change in recognizing positive and neutral [effect of group, *F*(1,38) = 5.18, *p* = 0.029, $\eta_{p}^{2}=0.13;$ *F*(1,38) = 5.67, *p* = 0.022, $\eta_{p}^{2}=0.12,$ respectively], but not the negative images [*F*(1,38) = 2.59, *p* = 0.12, ns.]. No interaction of load with group was noted for any image type. This means that WM load application resulted in an equal decrease in hit rates and response bias shift toward more conservative for negative images in both participants in neutral state and under threat, while recognition of neutral and positive images remained overall more conservative in the threat group.

## Discussion

We examined the effect of WM load on incidental memory for emotional stimuli in two groups of participants: in neutral affective state and under threat of electric shock. The participants performed the *N*-back task at low- and high-WM load while an affective distracter image was briefly flashed within the central focus of attention in a random quarter of the trials, so as to prevent anticipation of distracter presentation.

In both groups, we observed that distracters slowed down the RTs and increased the error rates, suggesting that the images were perceived and interfered with the cognitive processing of the WM task. Although the participants under threat showed slightly faster RTs, the groups did not significantly differ in terms of task performance indicating that the threat manipulation did not have any significant impact on *N*-back task performance. Importantly, we did not find any differential effect in RTs for the three types of distracter images, indicating that emotional distracters displayed very briefly (250 ms) do not interfere with main task performance more than neutral images, in contrast with previous studies that used longer emotional image exposure (Kensinger and Corkin, 2003).

Furthermore, in both groups of participants we observed a similar enhancement of immediate recognition memory for negative and positive stimuli based on hit rates, reflecting the frequently reported advantage of emotional stimuli to enhance immediate memory (Cahill and McGaugh, 1998; Rozin and Royzman, 2001; Talmi et al., 2008; Mather and Sutherland, 2011). EEM was equally present in both groups in the LL condition, with stronger enhancement for negative pictures and no significant between-group difference. Thus, we did not observe a stronger negativity bias in immediate recognition memory in participants under threat of shock as would have been predicted by increased processing of mood-congruent stimuli (Mitte, 2008; Robinson et al., 2011).

### Load erases the advantage of emotional stimuli

We observed a reduction in the number of correctly recognized items (hit rate) in the memory test for pictures presented during the high WM load condition. Importantly, the reduction was observed for the emotional items but not for neutral ones suggesting that arousing pictures may have been easier to ignore under high WM load. The participants in the threat induction group overall had worse memory for the distracter pictures which is consistent with the observation that arousal (fear of electric shock, in this case) imposes a level of cognitive load that taxes WM and limits the processing of task-irrelevant items, albeit emotionally charged (Mather and Sutherland, 2011; Vytal et al., 2012).

The elimination of EEM due to WM load is in line with the arousal-biased competition in memory (Mather and Sutherland, 2011) that proposes that emotional arousal enhances memory for prioritized information at the cost of goal-irrelevant information, regardless of the perceptual or affective details of the prioritized item. In our experiments, the letters in the *N*-back task were the goal-relevant items and took priority over the emotional distracters when the task difficulty and load on the WM was high. As a result, emotional pictures were not remembered better than neutral pictures.

It is known that items that signal emotional relevance are more likely to be processed in the presence of competing distracters than non-emotional items (Öhman et al., 2001), and studies report that emotional memory enhancement is stronger when attentional resources are restricted (Kensinger and Corkin, 2004; Talmi et al., 2007). Yet, our result shows that their memory trace can be weakened when WM load during or soon after presentation is sufficiently high. Interestingly, our results indicate that not recognition memory in general but enhancement of memory for emotional images depends on WM capacity upon encoding since the recognition accuracy for neutral images was very similar in both load levels in both experiments (Table 2; Figure 3).

Current evidence suggests that the immediate memory advantage of emotional items stems partly from enhanced attention and sensory processing (Vuilleumier, 2005; Talmi et al., 2008), with negative items being particularly likely to benefit from fast amygdala-mediated visual processing during short exposure times (Tamietto and de Gelder, 2010; Ritchey et al., 2011). However, some studies report that cognitive effort may lead to a reduction of memory. For example, Kensinger and Corkin (2004) found memory reduction for explicitly encoded negative words under certain circumstances – when the participants concurrently performed a difficult auditory discrimination task and the words were low-arousing. Most recently, Talmi and McGarry (2012) reported that the emotional memory enhancement can only be moderated to the level of neutral items when the latter are paid full attention to and emotional items are only partly attended in a dual-task condition. Our result is in line with these findings and further extends them showing that cognitive load leads to an overall reduction in recognition memory for negative visual scenes that are sparsely presented and encoded in a purely incidental manner. We thus conclude that application of WM load during arousing image viewing can attenuate the emotional bias in immediate recognition memory.

Emotional stimuli may be remembered better because they attract more attention upon encoding (Hamann, 2001). Such attentional enhancement could cause the negative memory enhancement that we observed in LL, but it cannot fully account for the result in HL where no emotional prioritization was observed even though the distracters significantly interfered with response times in both conditions. The result was also not driven by inattentional blindness in the HL condition because in all conditions the recognition rates corrected for guessing are well above chance level and the result pattern was replicated in both groups of participants. In fact, there is evidence that attention allocation is not the sole factor behind immediate memory enhancement (MacKay et al., 2004) and it has been suggested that under conditions of diminished attentional capacity due to concurrent cognitive load, distracters are perceived automatically and involuntarily (Lavie, 2000). Taken together, it is unlikely that the observed attenuation of emotional bias in recognition memory is due to impaired visual processing of distracters at exposure caused by reduced perceptual capacity under HL. It may rather imply that load interrupts a later stage of processing of emotional information, such as short-term memory or immediate memory consolidation, leading to deteriorated performance upon retrieval.

False alarm rates (Table 3) did not significantly differ between the two experimental groups, displaying a clear effect of emotional category, with highest scores for negative, followed by positive and neutral images. This result is consistent with previous reports showing a higher amount of false memories for emotional images (Gallo et al., 2009; Gallo, 2010), as well as a shift toward most liberal response bias for negative, followed by positive stimuli (Dougal and Rotello, 2007; Kapucu et al., 2008). In fact, all participants were most liberal in judging negative items as remembered, demonstrating that response bias plays a role in the EEM (Phelps and Sharot, 2008), also upon immediate testing.

A potential limitation of our study is the fact that in the mood-manipulation group we used the threat of shock paradigm without actually delivering the electric shock that could have resulted in a low or no anxiety in some participants. The subjective responses given by the participants on a Likert scale is a modest proof of anxiety as it is difficult to judge whether the average score (2.76) indicates a sufficiently high level of experienced anxiety, nor can a verifying comparison of this result be made, as no measurement of anxiety was taken in the neutral experimental group.

## Conclusion

In conclusion, we have demonstrated that a challenging cognitive task employing WM load can diminish the formation of incidental emotional memories, presumably by (1) exhausting the cognitive resources that could otherwise be captured by salient arousing distracters, and (2) shifting focus so that all distracters, emotional, or neutral, become goal-irrelevant. Notwithstanding the non-difference between the two experimental groups, we have found a very robust effect of load on incidental emotional memory, and suggest that engaging in a neutral task that loads the WM, especially in situations of stress and anxiety, can be a method to not only provide distraction from current emotional state (Van Dillen and Koole, 2007) but also to tune down future negative memories of perceived events.

## Conflict of Interest Statement

The authors declare that the research was conducted in the absence of any commercial or financial relationships that could be construed as a potential conflict of interest.

## Footnotes

^1^According to user legal agreement, the use of IAPS images is not permitted by for-profit institutions. To conduct psychological experiments with affective stimuli, a new set of controlled images has been developed.

^2^Due to data acquisition problems the *N*-back data of three participants in experimental group 1 (no-threat) could not be analyzed.

^3^Due to data acquisition problems the memory scores of two participants in experimental group 1 (no-threat) could not be analyzed.
